# Fisher–Shannon Analysis of Sentinel 1 Time Series from 2015 to 2023: Revealing the Impact of *Toumeyella Parvicornis* Infection in a Pilot Site of Central Italy

**DOI:** 10.3390/e27070721

**Published:** 2025-07-03

**Authors:** Luciano Telesca, Nicodemo Abate, Michele Lovallo, Rosa Lasaponara

**Affiliations:** 1 Institute of Methodologies for Environmental Analysis, National Research Council, 85050 Tito, Italy; rosa.lasaponara@cnr.it; 2 Institute of Cultural Heritage, National Research Council, 85050 Tito, Italy; nicodemo.abate@cnr.it; 3 ARPAB (Agenzia Regionale per la Protezione dell’Ambiente della Basilicata), 85100 Potenza, Italy; michele.lovallo@arpab.it

**Keywords:** Sentinel 1, vegetation, Fisher–Shannon analysis

## Abstract

This study investigates the capability of Sentinel-1 (S1) SAR time series to identify vegetation sites affected by pest infestations. For this purpose, the statistical method of the Fisher–Shannon analysis was employed to discern infected from unifected forest trees. The analysis was performed on a case study (Castel Porziano) located in the urban and peri-urban areas of Rome (Italy), which have been significantly impacted by *Toumeyella parvicornis* (TP) in recent years. For comparison, the area of Follonica (Italy), which has not yet been affected by this insect, was also analyzed. Two polarizations (VV and VH) and two orbit types (Ascending and Descending) were analyzed. The results, supported by Receiver Operating Characteristic (ROC) analysis, demonstrated that VH polarization in the Descending orbit provided the best performance in identifying TP-infected sites.

## 1. Introduction

Insect-driven disturbances represent one of the most pressing threats to global forest health, particularly affecting vulnerable ecosystems such as Mediterranean pine forests, boreal coniferous stands, and subtropical broadleaf plantations. These forest types are often characterized by low species diversity, homogeneous canopy structure, and high exposure to climatic stressors, making them especially susceptible to large-scale infestations and rapid canopy degradation [1,2].

The ecosystem services they provide—such as carbon sequestration, soil stabilization, biodiversity support, and recreational and cultural value—are critically compromised when infestations occur. The degradation of forest structure and function caused by pests not only results in direct biomass loss but also accelerates cascading effects on hydrological regulation and habitat integrity [3,4].

In this context, Earth Observation (EO) technologies play a vital role in identifying, mapping, and monitoring pest-induced disturbances across wide spatial and temporal scales. While optical EO approaches have proven effective in detecting vegetation stress and mortality [5,6], Synthetic Aperture Radar (SAR) systems offer unique advantages, particularly under persistent cloud cover or low-light conditions.

SAR-based analyses are seeing a rise in application for various purposes, including the following: (i) deforestation and forest degradation monitoring [7,8]; (ii) identifying key drivers behind forest changes [9]; (iii) mapping forest fire events and assessing fire severity [10,11]; (iv) evaluating damage from extreme weather events or extended droughts [12,13]; (v) characterizing forest seasonality and phenology [14]; and (vi) classifying vegetation types and tracking forest type changes and losses [15,16,17]. However, the use of SAR time series to detect biotic stress still remains underexplored, especially with regard to non-invasive, statistically robust methodologies capable of capturing subtle structural and phenological shifts.

In fact, SAR data can detect alterations in vegetation structure and moisture content, which are indicative of insect infestations or disease. These modifications manifest as variations in the backscatter signal and can be analyzed to identify impacted areas.

*Toumeylla parvicornis* (TP) is an insect species native to the Americas that has become invasive in Italy, where it has been spreading since 2015, particularly affecting *Pinus pinea* trees [18,19,20]. TP produces large quantities of honeydew, which gives the affected trees a glossy appearance. This creates an ideal environment for the growth of sooty mold, which coats the pine needles and branches. As the mold accumulates, it reduces photosynthetic activity, leading to the decline and eventual death of the trees. The most flourishing moment for the metabolic and reproductive activity of this parasite begins in the spring months and ends in the summer, with a period of inactivity between autumn and winter. Nevertheless, due to the rise in average global temperatures, an increase in the activity of this parasite has been noted throughout the year [21,22]. This resulted in widespread tree desiccation, which often precedes the death of affected individuals in the following years [23].

## 2. Data

**Figure 1:**
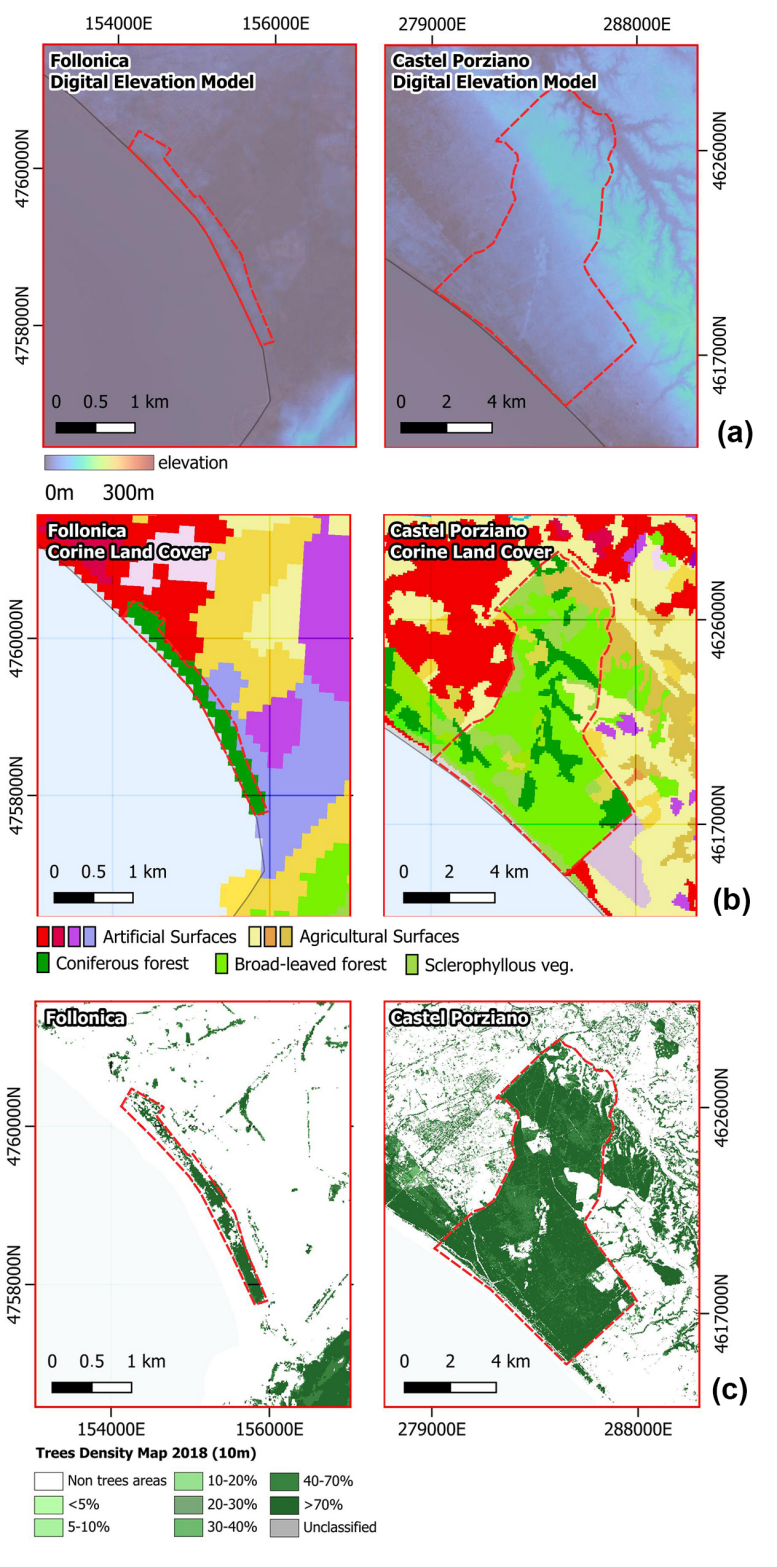
(**a**) Digital elevation model from SRTM (Shuttle Radar Topography Mission) from GEE; (**b**) Corine land cover 2018 from GEE; (**c**) tree density map from [25].

The present study utilizes the Sentinel-1 VV and VH polarization time series (2015–2023) for two types of orbit (Ascendent and Descendent) available through Google Earth Engine for monitoring vegetation dynamics in a peri-urban area of Rome. The focus area, the Presidential Estate of Castel Porziano, spans approximately 6039 hectares and is located 25 km southeast of Rome (Figure 1). Castel Porziano was chosen due to its significant impact from TP infestations in recent years [22]. In fact, in 2020, the TP infestation, exacerbated by climate change, reached a peak in the region, causing extensive damage to *Pinus* species. To assess the discrimination capability of SAR data, we selected Follonica as a control site. This location was chosen because its geographical characteristics are very similar to those of Castel Porziano. In particular, it is a coastal pine forest that extends for about 150 hectares, located along the same coastline, even if about 200 km away. Both areas share similar plant species and coastal exposure and, according to the Köppen climate classification [24], fall into the same climate category, in particular the Cs group (temperate climates with dry summers). It shares the same *Pinus pinea* L. (stone pine) species but had no recorded TP infestation as of 2023 (Figure 1). Furthermore, the two sites are monitored by the Forestry, Environmental and Agri-Food Unit Command of the Italian Carabinieri. At the end of 2023, the only critical issue and difference between the two sites was the infestation of TP in Castel Porziano. The classification between infected (CP) and uninfected (Fol) is supported by the publicly available documentations provided by the National Phytosanitary Service of Italy (https://www.protezionedellepiante.it/ (accessed on 26 June 2025).

For the two sites, 150 sample points were selected, respectively, in the area indicated as coniferous forest by Corine Land Cover (Figure 1c). At these points, data were sampled along the entire time series considered 2015-2023 for a total dataset, per site, of about 270 images. The spatial resolution of Sentinel-1 pixels, according to Google Earth Engine, is 10 m, while the data were sampled with a sampling time of 12 days. Sentinel-1 data were used in the VV and VH bands, the two polarization available in GEE, pre-processed with Sentinel1 Toolbox as follows: (i) removal of thermal noise, (ii) radiometric calibration, and (iii) terrain correction using SRTM 30 or ASTER DEM for areas above 60 degrees latitude, where SRTM is not available. The final terrain-corrected values are converted to decibels using the logarithmic scale. The data are characterized by a small percentage of gaps (see Table 1) that do not affect the results.

## 3. Method

The Fisher–Shannon method is based on computing the Fisher Information Measure (FIM) and Shannon entropy (SE), which characterize, respectively, the local and global characteristics of the probability density function of the series. FIM quantifies order and organization within the series [26], whereas SE serves as an indicator of uncertainty [27]. Their mathematical definitions are as follows:

(1) $$FIM=\int_{-\infty }^{+\infty }{\left(\frac{\partial }{\partial x}f\left(x\right)\right)}^{2}\frac{dx}{f(x)}$$

(2) $$SE=\int_{-\infty }^{+\infty }f_{X}\left(x\right)\log\left(f_{X}\left(x\right)\right)dx$$

where *f*(*x*) represents the probability distribution function of the series *x*. Instead of SE, it is often more practical to use the Shannon entropy power *N_X_*, which is always positive and defined as follows:

(3) $$N_{X}=\frac{1}{2\pi e}e^{2SE}.$$

To reliably estimate FIM and $N_{X}$, a kernel-based estimation technique is employed [28]. The probability density function f(x) is estimated using the following formula [29,30]:

(4) $$\hat{f}\left(x\right)=\frac{1}{Mb}\sum_{i=1}^{M}K\left(\frac{x-x_{i}}{b}\right),$$

where *M* is the length of the time series, *b* is the bandwidth, and K(u) is the kernel function. The kernel K(u) is a continuous, symmetric, and non-negative function that satisfies the following conditions:

(5) $$\int_{-\infty }^{\infty }K\left(u\right)\;du=1,\;\mathrm{and}\;\int_{-\infty }^{\infty }u\;K\left(u\right)\;du=0.$$

The estimation of f(x) is optimized through an integrated method based on the algorithms proposed by Troudi et al. [31] and Raykar and Duraiswami [32], using a Gaussian kernel of the following form (The algorithm outline is provided in the Supplementary Materials):

(6) $$K\left(u\right)=\frac{1}{\sqrt{2\pi }}e^{-u^{2}/2}.$$

The Receiver Operating Characteristic (ROC) analysis is used tool for evaluating classifier performance. In binary classification, instances are categorized as either “positive” or “negative,” with a classifier assigning them to predicted classes [33]. Depending on the classification outcome, four possible scenarios arise as follows: a *True Positive* occurs when a positive instance is correctly identified, a *False Negative* when a positive instance is misclassified as negative, a *True Negative* when a negative instance is correctly identified, and a *False Positive* when a negative instance is mistakenly classified as positive [33].

The classifier’s performance can be quantified using the *True Positive Rate* (TPr) and the *False Positive Rate* (FPr), defined as follows:

(7) $$TPr=\frac{Number\;of\;True\;Positives}{Total\;Positives},$$

(8) $$FPr=\frac{Number\;of\;False\;Negatives}{Total\;Negatives}.$$

The ROC curve illustrates the relationship between TPr (on the y-axis) and FPr (on the x-axis) depending on a threshold *T*. In ROC space, the point (0, 1) represents a perfect classifier, while the diagonal line given by y=x corresponds to random classification. Each point on the ROC curve reflects a trade-off between TPr and FPr for a specific threshold. The optimal classification threshold is often chosen as the point closest to (0, 1). Additionally, classifier performance is frequently quantified using the Area Under the ROC Curve (AUC), with a larger AUC indicating better classification performance.

## 4. Results

We analyzed 150 pixel time series in each of the two investigated sites. Although there may be variability in the time dynamics among the individual pixels, the sufficiently large number of pixels in each area allows for a statistical analysis based on the Fisher–Shannon method and ROC analysis that could ensure that the average behavior of the two areas is adequately represented.

For each pixel time series, we computed the FIM and $N_{X}$. Although the data contain a small percentage of gaps, this does not affect the results, as both informational quantities are derived from the data distribution f(x).

**Figure 2:**
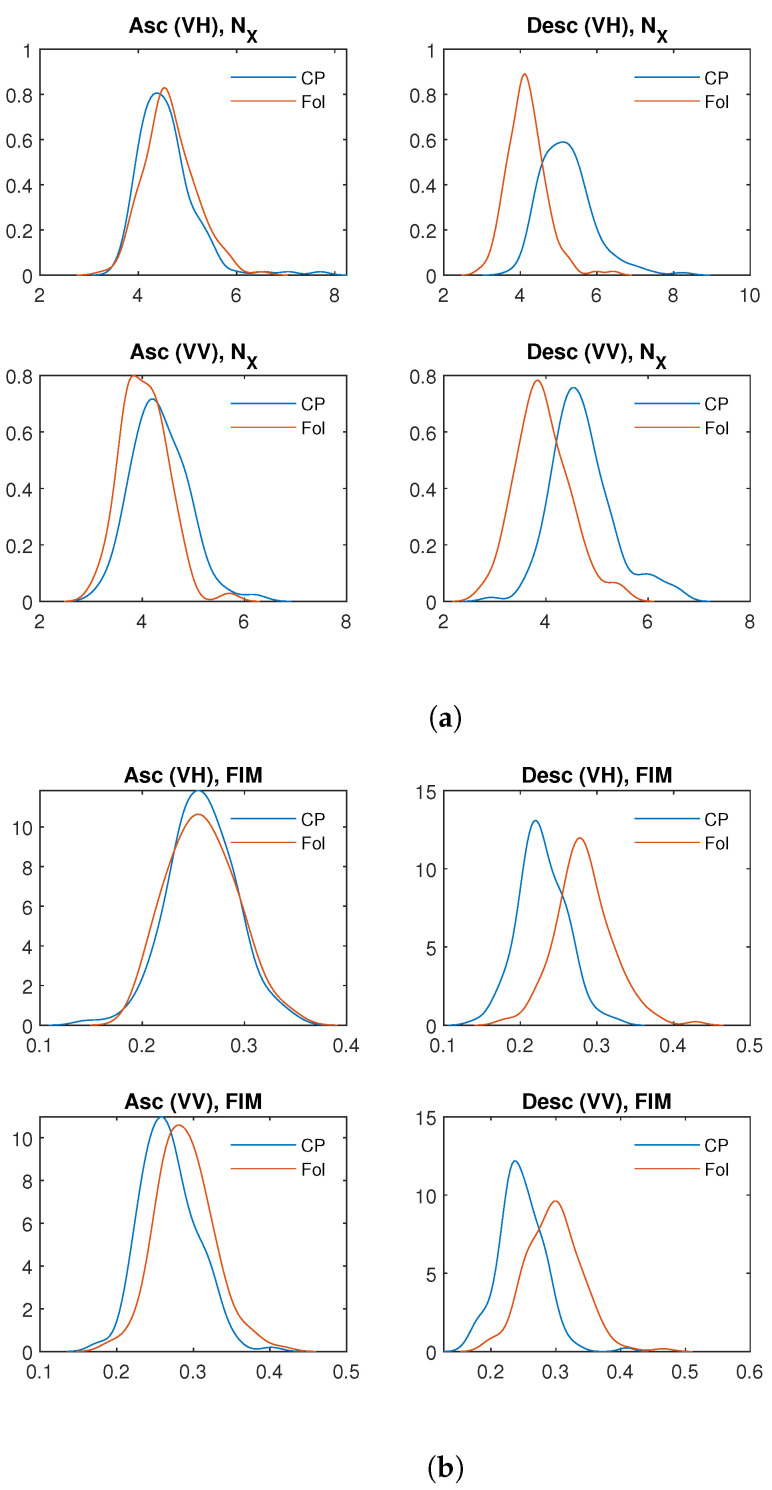
Distribution of (**a**) $N_{X}$ and (**b**) FIM values of the analyzed data.

We analyzed the Receiver Operating Characteristic (ROC) curves for both FIM and $N_{X}$ to evaluate their effectiveness in distinguishing between infected and non-infected sites. ROC curves are a widely used tool for assessing the performance of binary classifiers, as they illustrate the trade-off between the true positive rate (TPr) and the false positive rate (FPr). The construction of the ROC curve follows a systematic procedure. As an example, let us consider the case of $N_{X}$ for descending orbit in the VH polarization. First, all values of $N_{X}$ are sorted in ascending order. A threshold *T* is then chosen within the range defined by the minimum and maximum. Given that the average $N_{X}$ value tends to be higher for infected pixels (Castel Porziano site) compared to uninfected ones (Follonica site) (Figure 2), a value from an infected pixel is considered a true positive (TP) if it lies above the threshold *T*, and a false negative (FN) if it lies below *T*. Conversely, a value from an uninfected pixel is counted as a false positive (FP) if it is above the threshold, and a true negative (TN) if it is below. By applying this classification for each possible value of *T*, the true positive rate (TPr) and false positive rate (FPr) can be calculated, producing a single point on the ROC curve. Repeating this process across the entire threshold range yields the complete ROC curve. The optimal threshold corresponds to the point on the ROC curve that is closest to the ideal coordinate (0, 1), representing maximum sensitivity and minimum false positive rate.

**Figure 3:**
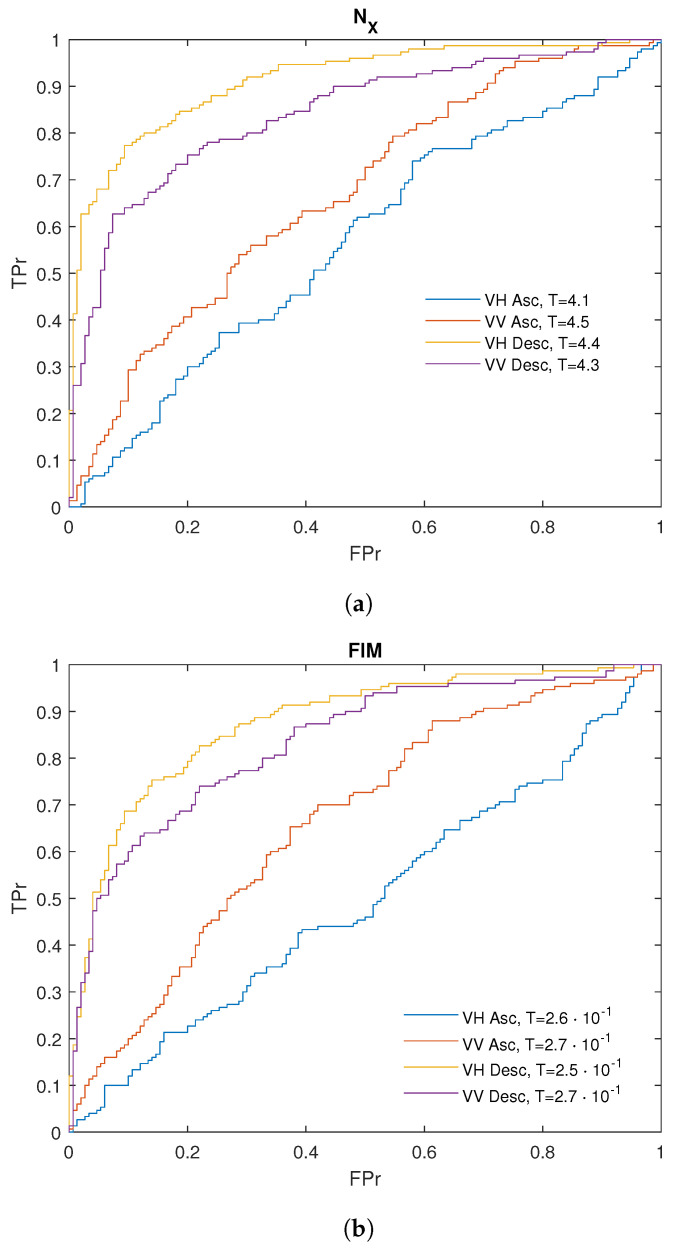
ROC curves of (**a**) Shannon entropy power and (**b**) Fisher Information Measure.

**Figure 4:**
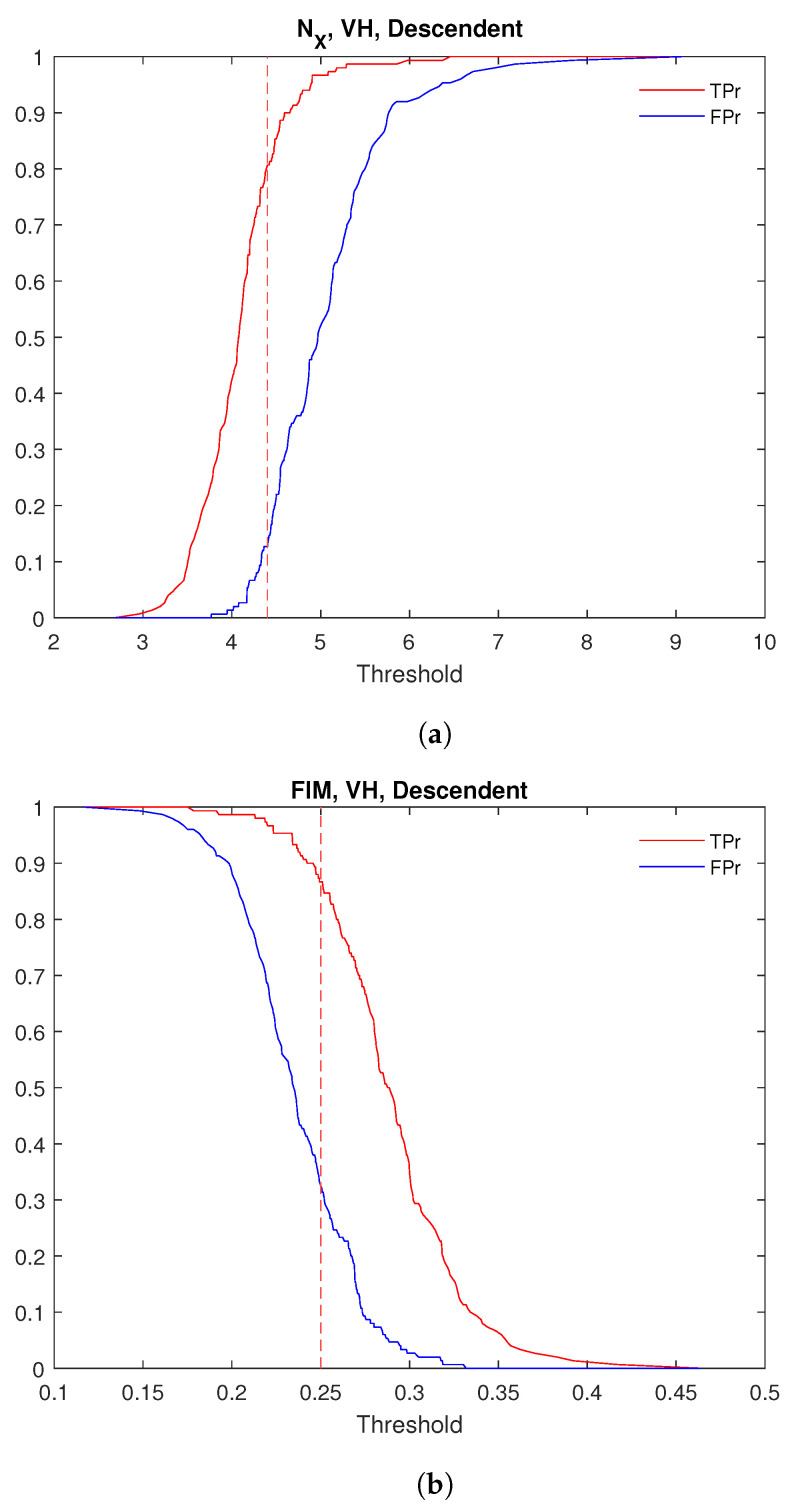
TPr and FPr versus threshold for (**a**) Shannon entropy power and (**b**) Fisher Information Measure for VH polarization in descendent orbit. The red dashed vertical line represents the optimal threshold.

**Figure 5:**
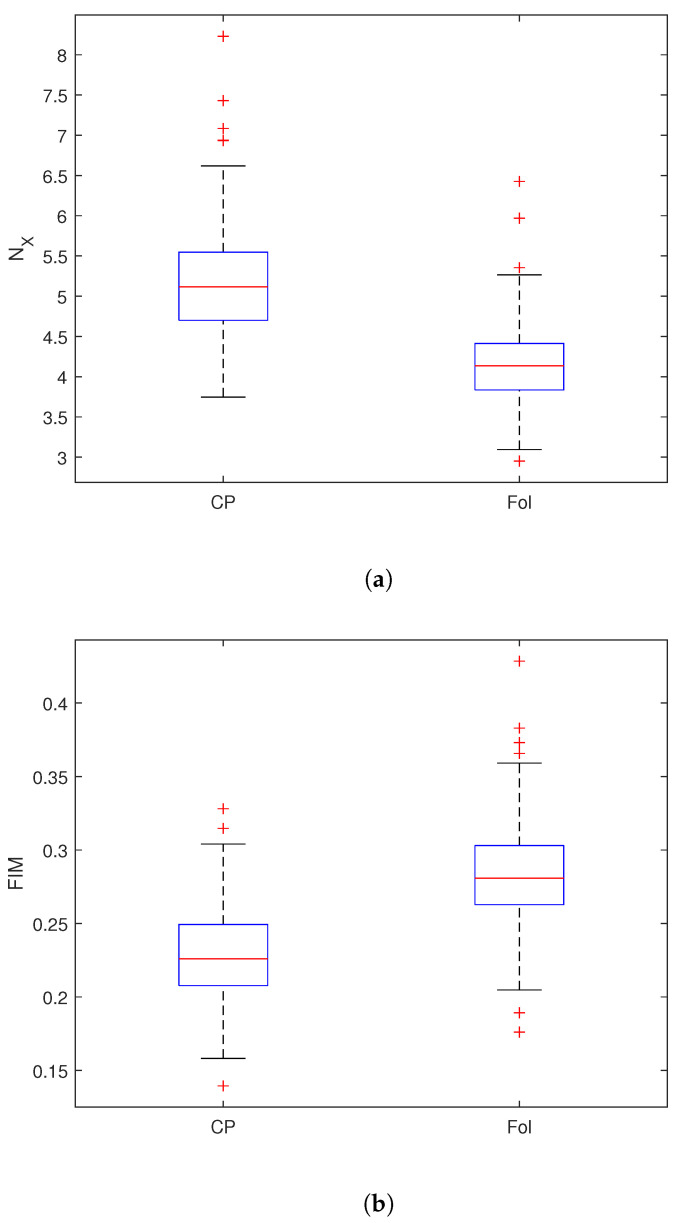
Boxplot of (**a**) Shannon entropy power and (**b**) Fisher Information Measure for VH polarization in descendent orbit.

Figure 3 shows the ROC curves for the Shannon entropy power $N_{X}$ (Figure 3a) and the FIM (Figure 3b). For both quantities, the ROC curves corresponding to the descending orbit in VH polarization lie above the others, indicating that this configuration provides the best performance for distinguishing between infected and uninfected pixels. Table 2 and Table 3 show the values of the ROC analysis for $N_{X}$ and the FIM of the analyzed data. Concerning $N_{X}$, the AUC reaches its maximum value (≈$9\cdot 10^{-1}$) for VH polarization in the descending orbit. Similarly, for FIM, the highest AUC (≈$8.8\cdot 10^{-1}$) is also observed for VH polarization in the descending orbit. Figure 4a illustrates the variation in TPr and FPr with the threshold. As shown, increasing the threshold for $N_{X}$ improves the detection of infected pixels (higher TPr) but also increases the misclassification of healthy pixels (higher FPr). Conversely, for FIM, decreasing the threshold enhances infected pixel detection but similarly raises the misclassification rate of healthy pixels. Thus, selecting the optimal threshold is always a trade-off between these two metrics. Typically, the threshold is chosen to maximize TPr while keeping FPr as low as possible to ensure a balanced and effective classification. The optimal discrimination threshold for $N_{X}$ is 4.4, resulting in a true positive rate (TPr) of 80% and a false positive rate (FPr) of approximately 13%. This outcome indicates that $N_{X}$ serves as an excellent discriminator between the two areas, effectively distinguishing between “infected” and “healthy” trees. The optimal threshold for FIM is 0.25, yielding a true positive rate of approximately 83% and false positive rate of 22% (Table 3). Figure 5 displays the boxplot for $N_{X}$ and FIM in the VH polarization for the descending orbit, demonstrating their optimal performance in distinguishing between Castel Porziano and Follonica. We applied the Wilcoxon rank-sum test and obtained *p*-values of approximately $10^{-35}$ for $N_{X}$ and $10^{-29}$ for FIM, indicating a highly significant difference between the medians of the two groups.

On average, TP-infected sites are characterized by a higher $N_{X}$ and a lower FIM than healthy sites. Since FIM and $N_{X}$ correspond, respectively, to the local and global characteristics of the distribution of SAR signals, the higher $N_{X}$ observed in TP-infected sites indicates that their distribution is primarily driven by global variations. In contrast, the distribution of SAR signals in healthy sites appears to be more influenced by local fluctuations. The observed difference in Fisher–Shannon response between infected and uninfected trees may be associated with variations in the photosynthetic activity. The *Pinus pinea* canopy in healthy conditions exhibits a well-defined seasonal pattern, which is effectively captured by the SAR signal, reflecting the order and organization within the time series. In contrast, TP infestation reduces photosynthetic activity, leading to widespread tree desiccation and a loss of phenological cycles. This results in a diminished seasonality and increased disorder in the SAR signal. The two adopted metrics effectively highlight both healthy and unhealthy vegetation conditions. Therefore, the classification of FIM and SE metrics within the SAR time series enhances the ability to discriminate alterations in vegetation structure and moisture content induced by insect infestations or diseases.

## 5. Discussion

VH polarization is particularly sensitive to volumetric scattering, resulting from the interaction of the radar signal with vertical vegetation elements such as branches, needles, and small twigs—components most affected by structural degradation caused by *Toumeyella parvicornis* infestation. Alterations like needle loss, reduced foliage moisture, and sooty mold formation significantly modify the canopy’s structure and dielectric properties, impacting the received SAR signal. In contrast, VV polarization is more influenced by surface reflections and is less effective in capturing subtle structural changes.

Orbital geometry further influences detection capability. The descending orbit, due to its different incidence angle, offers a more favorable configuration for observing the structural changes typical of coastal pine forests. This alignment may enhance interaction with the vertical architecture of the *Pinus pinea* canopy, improving sensitivity to infection-driven changes.

From an information-theoretic perspective, infected sites exhibit higher $N_{X}$ values and lower FIM values compared to healthy ones. This pattern reflects greater signal variability (disorder) and reduced local organization, consistent with disrupted phenological cycles. Conversely, uninfected sites maintain more regular SAR temporal signatures, indicative of stable canopy conditions. These differences, confirmed by ROC analysis, are physically grounded in radar–vegetation interaction mechanisms and in the structural effects of infestation. The superior performance of the descending VH configuration highlights its robustness in detecting vegetation degradation.

The transferability of the Fisher–Shannon framework developed in this study is supported by both its theoretical basis and the biophysical nature of SAR responses. Although applied here to *T. parvicornis* infestation in *P. pinea*, the approach does not rely on species-specific traits. Instead, it uses two general descriptors—FIM and $N_{X}$—which quantify local order and global variability in SAR time series. These metrics are well suited to monitoring disturbances that affect canopy structure, dielectric behavior, or phenological regularity, making the method applicable to a broad range of forest stressors, including pests like *Ips typographus*, *Lymantria dispar*, and *Sirex noctilio*, or fungal dieback.

The methodology is also sensor-agnostic. While this study uses C-band Sentinel-1 data due to its accessibility and revisit frequency, the same framework can be applied to L-band systems like ALOS-2 or the forthcoming NISAR mission. L-band’s longer wavelength provides deeper canopy penetration and is more sensitive to sub-canopy changes, potentially improving early detection of stress in denser or mixed forests. Sensor transfer may require tuning the time series resolution and kernel bandwidths used for density estimation, but the overall workflow—comprising time series extraction, entropy and FIM computation, and ROC evaluation—remains consistent. Given its non-parametric, generalizable nature and proven sensitivity to vegetation change, the method is highly transferable across ecosystems, disturbance types, and SAR platforms.

However, the ROC analysis presented in our study does not incorporate cross-validation, which may lead to an overestimation of model performance due to potential overfitting. Thus, the reported AUC values may not generalize well to independent datasets, and future work should address this through more robust validation approaches.

## 6. Conclusions

This study investigates the potential of Sentinel-1 (S1) data for monitoring and detecting forest vegetation infestations and insect-related diseases, focusing on the following two test sites in Italy: Castel Porziano, which is affected by Toumeyella parvicornis, and Follonica, which remains unaffected. To date, the only known difference in vegetation health between these sites is the presence of the parasite. The results demonstrate a strong impact of the parasite on the Sentinel-1 SAR signal, which can be directly correlated with vegetation changes caused by the following: (i) drying and reduced humidity in the canopy structure, and (ii) a progressive decline in canopy density due to the inhibited production of new needles [21,22]. This impact is clearly visible through the statistical approach used in this study.

The application of ROC analysis of the Fisher–Shannon-based metrics enabled the assessment of the performance of VV and VH polarizations for two orbit types (Ascending and Descending). The best discrimination performance was achieved with VH polarization in the descending orbit, since this polarization is more sensitive to changes in canopy density and the dielectric constant [34,35]. Results from our investigations clearly demonstrate that S1 data can effectively capture changes in vegetation structure and moisture content associated with insect infestations or diseases, enhancing the detection of changes in the backscattering signal and capturing deviations from the expected behavior. This enables the clear discrimination between healthy and unhealthy areas.

Additional investigations will be conducted to further explore the potential and limitations of Sentinel-1 data. However, the significance of these preliminary results lies in the demonstration that early detection of infestations is critical for defining mitigation strategies and effectively counteracting rapid spread.

## Figures and Tables

**Table 1:** Percentage of the mean number of missing data per pixel.

	Castel Porziano	Follonica
VH (Asc)	0.03%	0.03%
VV (Asc)	0.03%	0.06%
VH (Desc)	0.07%	0.06%
VV (Desc)	0.07%	0.06%

**Table 2:** Results of the ROC analysis for $N_{X}$. The asterisk (*) refers to the value of TPr and FPr corresponding to the optimal threshold.

	VH (Asc)	VV (Asc)	VH (Desc)	VV (Desc)
AUC	$5.7\cdot 10^{-1}$	$6.7\cdot 10^{-1}$	$9.1\cdot 10^{-1}$	$8.4\cdot 10^{-1}$
Optimal threshold	4.1	4.5	4.4	4.3
TPr *	$6.1\cdot 10^{-1}$	$5.8\cdot 10^{-1}$	$8.0\cdot 10^{-1}$	$7.5\cdot 10^{-1}$
FPr *	$4.8\cdot 10^{-1}$	$3.3\cdot 10^{-1}$	$1.3\cdot 10^{-1}$	$2.0\cdot 10^{-1}$

**Table 3:** Results of the ROC analysis for FIM. The asterisk (*) indicates the values of TPr and FPr corresponding to the optimal threshold.

	VH (Asc)	VV (Asc)	VH (Desc)	VV (Desc)
AUC	$4.9\cdot 10^{-1}$	$6.6\cdot 10^{-1}$	$8.8\cdot 10^{-1}$	$8.3\cdot 10^{-1}$
Optimal threshold	$2.6\cdot 10^{-1}$	$2.7\cdot 10^{-1}$	$2.5\cdot 10^{-1}$	$2.7\cdot 10^{-1}$
TPr *	$4.3\cdot 10^{-1}$	$6.5\cdot 10^{-1}$	$8.3\cdot 10^{-1}$	$7.4\cdot 10^{-1}$
FPr *	$3.9\cdot 10^{-1}$	$3.7\cdot 10^{-1}$	$2.2\cdot 10^{-1}$	$2.2\cdot 10^{-1}$

## Data Availability

The data presented in this study are openly available in Google Earth Engine https://earthengine.google.com/.

## Supplementary Materials

The following supporting information can be downloaded at: https://www.mdpi.com/article/10.3390/e27070721/s1.
