# Supplementary material for: Fisher–Shannon Analysis of Sentinel 1 Time Series from 2015 to 2023: Revealing the Impact of Toumeyella Parvicornis Infection in a Pilot Site of Central Italy
Source: Entropy (Basel). 2025 Jul 3;27(7):721. doi: 10.3390/e27070721 (PMC12294176; doi:10.3390/e27070721)
Supplement: Supplementary file 1 [file entropy-27-00721-s001.zip › entropy-3664514-supplementary.pdf]

Algorithm: BandwidthKernelGaussian

Input:

- X: dataset
- Nxe: number of evaluation points
- NomeFile, NomeDir, id: output path info

Output:

- Hout: optimal bandwidth
- S: Shannon entropy
- F: Fisher information

Steps:

1. Normalize the data X (zero mean, unit variance).
2. Estimate initial bandwidth H0 using Silverman-like rule.
3. Iteratively update the bandwidth H:
   a. Compute the second derivative estimate (r=2) of the density **using FastDerivativeGaussian**.
   b. Compute the functional Jf (energy of second derivative).
   c. Update bandwidth H using plug-in formula.
   d. Recompute the 0-th derivative (r=0) and check convergence of the density.
4. Once convergence is achieved:
   a. Evaluate density estimate over a fine grid Xe using final bandwidth.
   b. Normalize the estimated density (area under curve = 1).
   c. Compute Shannon entropy S:
      - $\int f(x) \log(f(x))\, dx$
   d. Compute Fisher information F:
      - $\int (f'(x))^2 / f(x)\, dx$
5. Write Xe and estimated density to output file.

Note: FastDerivativeGaussian computes a derivative of Gaussian kernel density estimate using optimized local sums.

Algorithm: FastDerivativeGaussian

Input:

- r : order of the derivative to compute (e.g., 0 for density, 2 for curvature)
- h : bandwidth
- Nxe : number of evaluation points (Xe)

Uses:

- Global variables: X (data), Xe (evaluation grid), N (number of data points)

Output:

- Yd: estimate of the r-th derivative of the density at points Xe

Steps:

1. Normalize X and Xe to [0,1] domain:
   - Compute global min/max of X and Xe
   - Rescale both X and Xe
   - Update bandwidth h accordingly

2. Compute prefactor:
   - q = coefficient involving (-1)^r / (sqrt(2π) * N * h^(r+1))

3. Estimate the required number of terms (P) in the series expansion:
   - Based on an accuracy threshold (eps), determine maximum expansion index P
   - Also compute integration range Rex depending on h and r

4. Construct local binning structure:
   - Divide domain into L = int(1/h)+1 bins (centered at spacing ~ h)
   - For each bin, store the start and end indices of data points within it

- This allows local evaluation (speed-up)

5. Precompute local kernel moments:

   - For each bin and each (p, r) index, precompute:

     Bl(bin, p, r) = Σ (x_i - center_bin)^p * ϕ(x_i - center_bin) * (x_i - center_bin)^r

   - Normalized by factorial(p-1)

6. Precompute coefficients matrix Ast(s, t) based on r and factorial expansions

7. For each evaluation point Xe(i):

   - Determine which bins influence Xe(i) (depending on Rex)
   - For each influencing bin:
     - Evaluate a truncated series expansion involving:
       - Ast(s, t) coefficients
       - Bl(bin, p, t) moments
       - Hermite-like terms depending on distance from bin center
   - Sum all contributions into Yd(i)

8. Rescale output by factor (original domain width)^(−(r+1))

Output:

- Yd: array of Nxe values representing the r-th derivative estimate at Xe

Note:

- This method exploits local expansions and symmetry of the Gaussian kernel for efficient computation.
